# Deep Transcranial Magnetic Stimulation: Improved Coil Design and Assessment of the Induced Fields Using MIDA Model

**DOI:** 10.1155/2018/7061420

**Published:** 2018-06-05

**Authors:** Amine M. Samoudi, Emmeric Tanghe, Luc Martens, Wout Joseph

**Affiliations:** Department of Information Technology, Ghent University-Imec, Ghent, Belgium

## Abstract

Stimulation of deep brain structures by transcranial magnetic stimulation (TMS) is a method for activating deep neurons in the brain and can be beneficial for the treatment of psychiatric and neurological disorders. To numerically investigate the possibility for deeper brain stimulation (electric fields reaching the hippocampus, the nucleus accumbens, and the cerebellum), combined TMS coils using the double-cone coil with the Halo coil (HDA) were modeled and investigated. Numerical simulations were performed using MIDA: a new multimodal imaging-based detailed anatomical model of the human head and neck. The 3D distributions of magnetic flux density and electric field were calculated. The percentage of volume of each tissue that is exposed to electric field amplitude equal or greater than 50% of the maximum amplitude of E in the cortex for each coil was calculated to quantify the electric field spread (V50). Results show that only the HDA coil can spread electric fields to the hippocampus, the nucleus accumbens, and the cerebellum with V50 equal to 0.04%, 1.21%, and 6.2%, respectively.

## 1. Introduction

Transcranial magnetic stimulation (TMS) is a noninvasive and painless method for activating neurons in the brain and can be used as a probe of higher brain functions and an intervention for neurological and psychiatric disorders [1]. Several coils were designed to stimulate different brain regions for different treatments (depression and Parkinson's disease) but, due to the electric field rapid attenuation deep in the brain, TMS has been restricted to superficial cortical targets, around 2-3 cm in depth [2]. However, recent studies show that treatment for depressions can also consider nonsuperficial brain areas of 3-5 cm depth [3], as well as deeper regions of 6-8 cm depth [4, 5].

Using traditional TMS, with circular or figure of eight (Fo8) coils, regions of deep brain cannot be reached, as the electric field decreased rapidly as a function of tissue depth for this type of coils [6]. Thus, much higher stimulation amplitudes were needed to stimulate deeper neuronal regions. However, such high intensities at the sources may raise many safety concerns and can cause local discomfort due to the direct activation of nerves and muscles in the scalp [7]. Coil designs suitable for deep TMS, such as double-cone coil [8], Halo coil [9], and H-coil [10] were developed to circumvent these limitations. The double-cone coil provides deeper field penetration and has been used to target the anterior cingulate cortex with the transsynaptic activation [11]. The Halo coil has been designed to increase the magnetic field at depth in the brain when used together with the existing Fo8 and circular coils typically used for TMS [9, 12]. The coil design will be a combination of two TMS coils mostly used to increase the deep penetration of the electric field: the double-cone coil and the Halo coil. Locations of activation in the brain are related to the area where the induced electric field is maximal. These locations, in turn, depend on the coils' placement and geometry as well as the head model for simulation studies [13]. Despite its importance and the increasing clinical use of the TMS coils, the knowledge of the spatial distribution of the induced electric field is not yet comprehensively investigated [14]. Different works investigated the induced electric field spatial distribution using experimental data or numerical simulations based methods with simplified head models such as spheres (i.e., [15, 16]) or human head models with very few tissues (i.e., [17, 18]). Recently, Deng et al. [15] published a comprehensive study using spherical human head model to quantify the electric field focality and depth of penetration of various TMS coils. However, considering the obvious and significant difference between the human brain geometry and the spherical form, the induced electric field distribution will be different in the two models. It is well understood that the structure of the brain, the resolution, and the number of tissues can affect the distribution of the electric field and the maximum electric field in the brain, which can result in wrongly identifying stimulation locations (i.e., [19] showed that the difference in electric field can be greater than 100 V/m between young and adult human head models). In the realistic head geometry and since the head surface is nonuniform and with a variable curvature, the resulting electric field distribution will be much more sensitive to the coil orientation and position [20]. Guadagnin et al. [14] recently published an extensive study providing a characterization of the induced E distributions in the brain of a realistic human model (Ella V1.3 from the Virtual population [21] containing consists of 76 different tissues in the whole body) due to various coil configurations. Recently, a new multimodal anatomical model of the human neck and head was developed by Iacono et al. [22]. The new high resolution model (up to 500 *μ*m) contains 153 structures in the head and the neck and provides detailed characterization of the deep brain tissues with an atlas-based segmentation, which makes the MIDA model among the most advanced image-based models for anatomical models in the state of the art.

The objective of this work is to use numerical models to design and investigate a combined deep TMS coil design using double-cone and Halo coils. Investigation of the brain model effect on the induced electric field was performed using the MIDA model. The novelty of this paper is as follows:

- Model a combined deep TMS coil consisting of Halo and double-cone coils to reach deep brain structures (hippocampus, the nucleus accumbens, and the cerebellum) and characterization of the induced electric field in the brain by the combined coil.
- Characterization of the induced electric fields using MIDA: among the most detailed state-of-the-art image-based anatomical models including validation of the simulations with experimental results.

## 2. Materials and Methods

### 2.1. Simulation Platform

TMS coils and human head model were modeled with a commercial software package Sim4Life [23]. This is a simulation platform, combining computable human phantoms with physics solvers and tissue models. Sim4Life provides a modern and user-friendly and contains state-of-the-art resources to allow a fast and easy experience when setting up model geometries. The magnetic flux density and the electric fields in the human head were analyzed with the Sim4Life magneto quasistatic solver, enabling the efficient modeling of quasistatic EM regimes by applying the finite element method on graded voxel meshes. The numerical simulations are based on the EM low frequency theory implemented in Sim4Life. For an electric field** E** and a magnetic field** B**, assuming a vector potential** A** with ∇  x  **A** = **B** and a scalar electric potential *φ*, the scalar potential equation is

(1) $$\begin{array}{c} \nabla .\tilde{\varepsilon }\nabla \varphi =j\omega \nabla .\tilde{\varepsilon }A \end{array}$$

$\tilde{\varepsilon \;}$ refers to the complex permittivity defined as $\tilde{\varepsilon \;}\;=\;\varepsilon +\;\sigma /j\omega$, *σ* is the electric conductivity, *ε* is the electric permittivity, and *ω* is the angular frequency. For a characteristic length *d* and a permeability value *μ*, the quasistatic approximation condition $\left|\omega^{2}\mu \tilde{\varepsilon }d^{2}\right|\ll 1$ ensures that the ohmic current only negligibly perturbs the B-field and the vector potential** A** is equivalent to the magneto-static vector potential **A**_0_. The static vector potential **A**_0_ can then be calculated by the Biot–Savart law (when *μ* is constant over the entire computational domain). Since most biological materials exhibit dielectric properties that obey *σ* ≫ *ωε* in low frequency, (1) can be simplified to

(2) $$\begin{array}{c} \nabla .\sigma \nabla \varphi =j\omega \nabla .\sigma A_{0} \end{array}$$

Equation (2) is implemented in the magneto quasistatic solver. All boundary conditions are neglected as zero Neumann boundary conditions, i.e., vanishing normal flux. The real-valued solver is used by this model. The electric field is calculated only in the lossy (*σ* ≠ 0) domain, whereas the H-field is calculated everywhere. Therefore, the default grid covers only the lossy domain.

### 2.2. Numerical Coil Models

**Figure 1:**
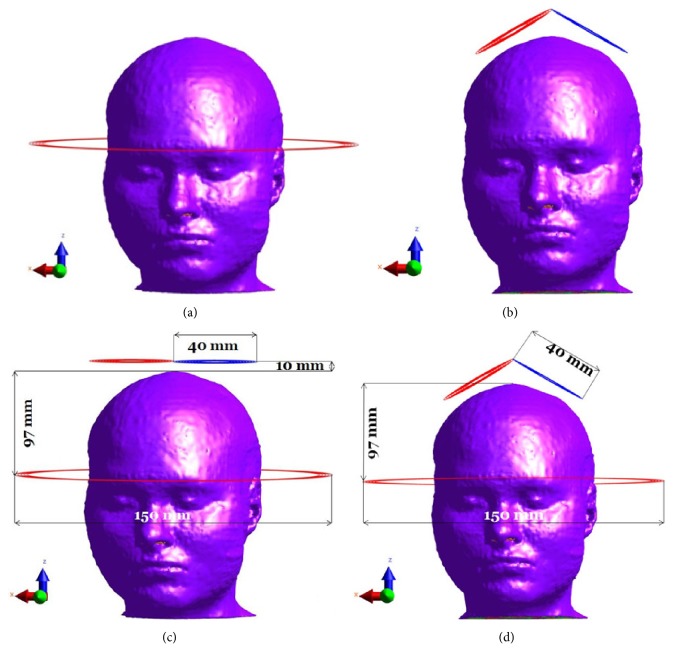
Overview of the magnetic coils and the MIDA head model. (a) Halo coil. (b) Double-cone coil. (c) HFA coil. (d) HDA coil.

New deep TMS coils were designed recently using combined coils. For example, Lu and Ueno [12] designed a combined coil consisting of Fo8 and Halo coils to reach deep brain structures. Since the double-cone coil is more considered for deep TMS [11], the coil design is the combination of the Halo coil with the double-cone coil to provide a deeper penetration of the electric field inside the brain structures.  Figure 1 shows the adult man (MIDA) head model with a Halo coil (Figure 1(a)), double-cone coil (Figure 1(b)), combined Halo and Fo8 coils (HFA) (Figure 1(c)), and HDA coil (Figure 1(d)). In order to compare the combined coil's performances with previous published TMS coils, we modeled the double-cone coil with two adjacent circular windings fixed at a 120° angle of 10 turns with inner and outer diameter of 15 mm and 40 mm, respectively, and the Halo coil with 5 circular windings of 150 mm and 138 mm, respectively [12]. The Fo8 coil is located 10 mm above the skin surface of the head to take into account the coil's insulation thickness and the Halo coil 97 mm below the head vertex [19]. Simulations were performed using pulse currents of 2.5 kHz frequency, based on the biphasic pulse frequency used by commercial TMS systems. We assumed a 100 % stimulator power output corresponding to 5 kA electric current in the coils [19]. The current flowing in the neighboring two wings of the Fo8 and the double-cone coils is in opposite directions. To assess the electric field distribution and spread in different brain tissues (gray matter, white matter, thalamus, hypothalamus, hippocampus, amygdala, nucleus accumbens, and cerebellum), the percentage of volume of each tissue exposed to an electric field amplitude equal or greater than half of the maximum amplitude of the electric field in the cortex for each coil was calculated (V50 used in [14]). The maximum of an amplitude distribution corresponds to its 99th percentile instead of the maximum to account for possible computational inaccuracies [24].

### 2.3. Anatomical Model and Tissue Dielectric Properties

**Figure 2:**
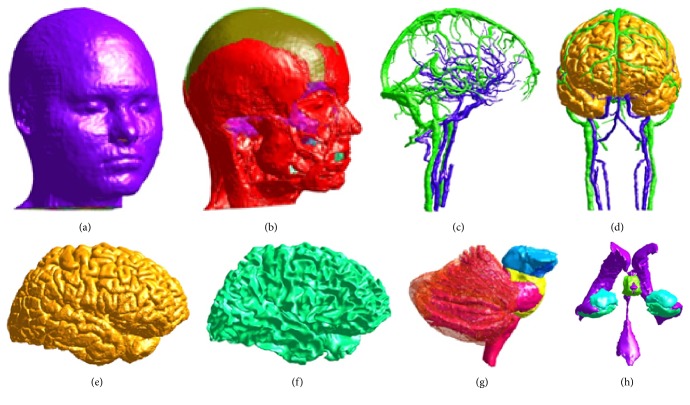
MIDA head model: model of a few representative structures of the head and neck. (a) Skin. (b) Muscles, the muscles are shown with the skull structures. (c, d) Vessels, the vessels are shown both without and with the GM. (e) GM. (f) WM. (g) Cerebellum and brainstem. (h) Ventricles, hippocampus, hypothalamus, and amygdala [22].

The MIDA human head model was used to investigate the coils' magnetic field interaction with brain tissue (Figure 2).

MIDA is among the most advanced multimodal imaging-based anatomical models of the human neck and head. The anatomical model comes with unique high resolution 153 structures, including several distinct deep brain structures, skull layers and bones, and nerves, as well as veins and arteries [22], which is highly relevant in our study to distinguish different deep brain structures and the induced electric fields within these brain tissues. The dielectric parameters of the tissues are set based on the database based on Gabriel et al. [25].

### 2.4. Validation: Simulation versus Experiments

**Figure 3:**
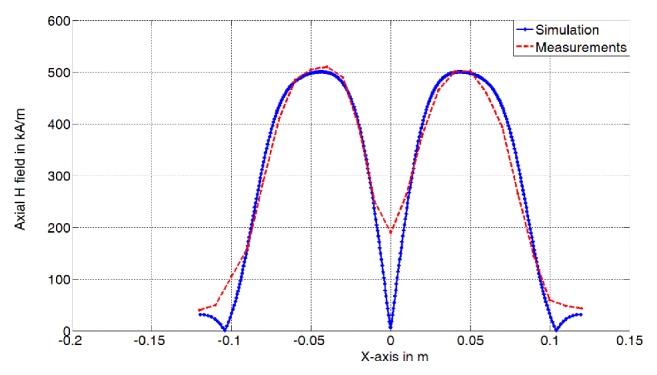
Axial component of the simulated and measured magnetic field (kA/m) at a distance of 20 mm, along the length of the TMS coil (Magstim 2nd Generation Double 70 mm remote control).

To validate the simulation software, we compared the numerical simulations of the magnetic fields of a commercial coil commonly used in the implementation of TMS with measurements from [22]. We considered the Double 70 mm Magstim 2nd Generation with remote control [25]. This coil is composed of 9 windings (inner and outer diameter of 32 mm and 48 mm, respectively). We considered a separation of 1 mm between the windings to take into account air gap and insulation.  Figure 3 shows the axial component of the simulated and measured magnetic field (kA/m) at a distance of 20 mm, along the TMS coil length. The calculations show good agreement with the measured field. Relative deviation of 0.12%-10.75% was obtained. We observe higher deviations at the center and the edges, which are due to the minor simplifications in the modeling of TMS coils.

## 3. Results and Discussions

### 3.1. B-Field Distribution

**Figure 4:**
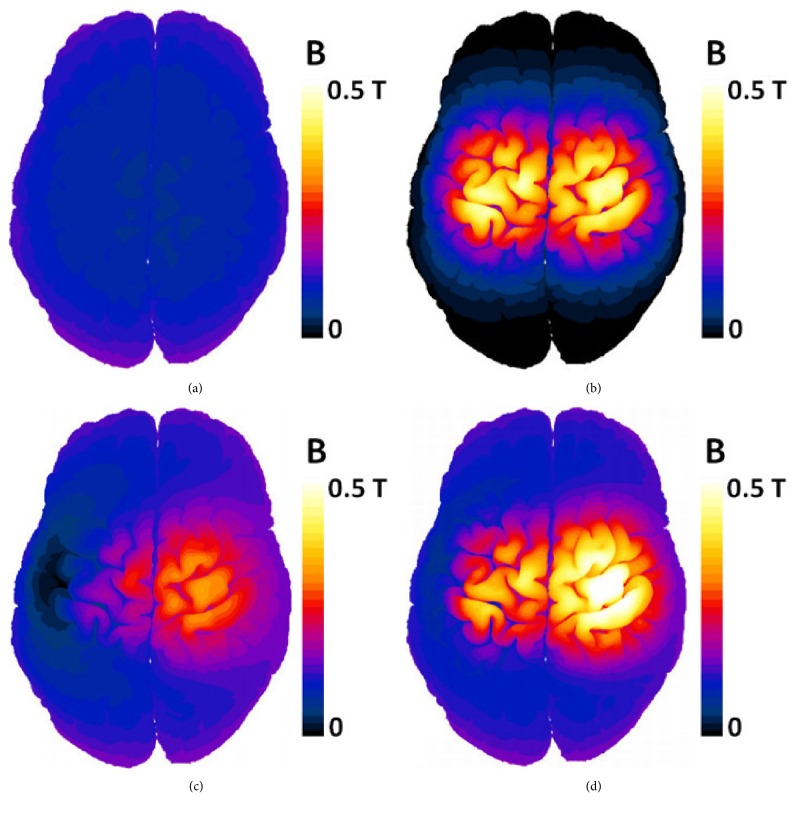
Magnetic flux density (absolute value in T) calculated in the gray matter of MIDA model for different coils. (a) Halo coil. (b) Double-cone coil. (c) HFA coil. (d) HDA coil.

Figure 4 shows the magnetic flux density on the surface of gray matter (GM) of the MIDA head model for Halo (a), double-cone (b), HFA (c), and HDA (d) coils for an equal separation of 10 mm and equal current applied to the two coils. It was observed that the maximum magnetic flux occurred near the coils and decayed quickly with distance from the coils for all the configurations. Higher values of B-field in brain were present in the right side as the HDA and the HFA coil were applied (Figures 4(c) and 4(d)). Comparison between the double-cone (Figure 4(b)) and the HDA (Figure 4(d)) configurations shows that adding the Halo coil resulted in a B-field decrease in the left hemisphere in favor of the right hemisphere. This is due to the fact that combining the Halo coil with the double-cone or Fo8 coils results in one side (right side) of the head being exposed to positive current from the two coils and the other side (left side) to positive and negative current from the coils. This effect will result in an increased field penetration in the right hemisphere when the HFA and the HDA coil operate. This asymmetric effect can also be trigged in the favor of the left hemisphere if we inverse the current direction in the double-cone or the Fo8 coils.

### 3.2. Electric Field Distribution

**Figure 5:**
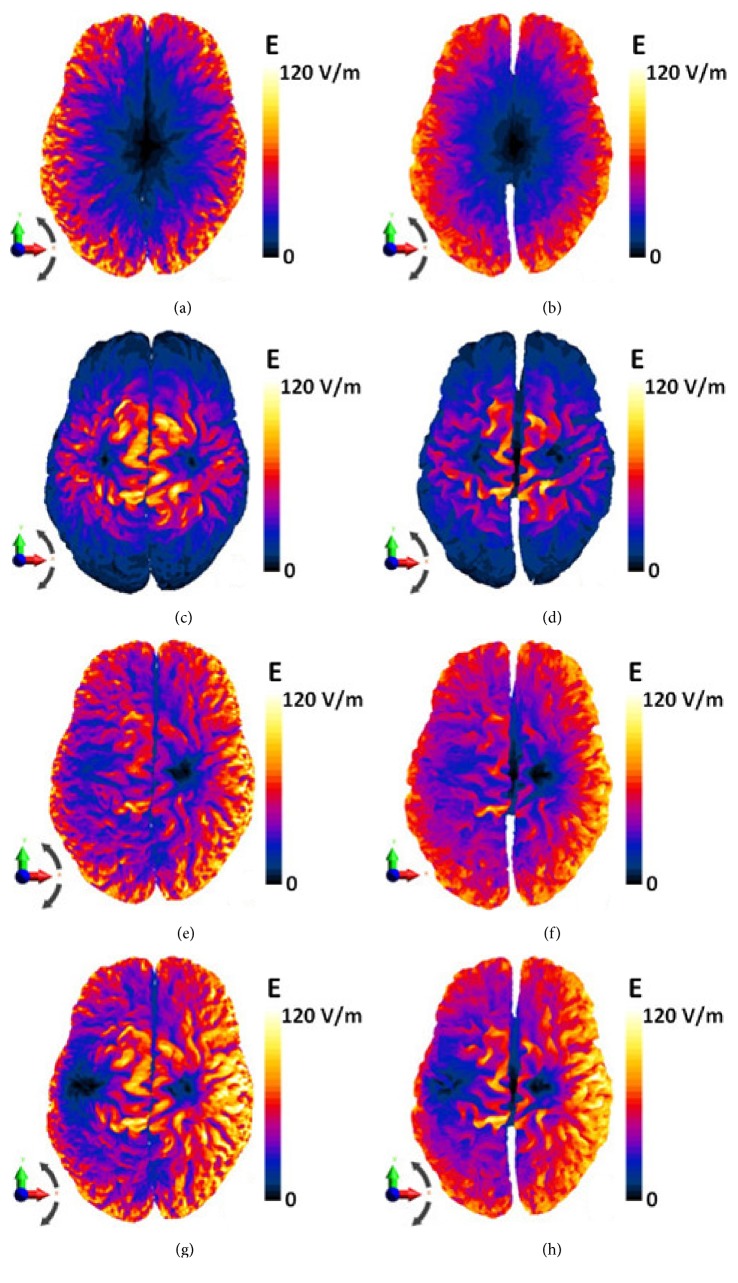
Electric field (absolute value in V/m) distribution in the GM (left column) and the WM (right column) for different coils. (a, b) Halo coil. (c, d) Double-cone coil. (e, f) HFA coil. (g, h) HDA coil.

Figure 5 shows the electric field spatial distribution on the brain gray matter and white matter for each TMS coil. For the Halo coil, the electric field was mainly produced in the periphery of the GM (Figure 5(a)) and the WM (Figure 5(b)) due to the proximity of this region to the Halo coil. Figure 5(a) of the GM shows slightly higher E-amplitudes than in Figure 5(b) of the WM, which can result in larger volume of tissue exposed to higher amplitudes of the electric field. Higher values of electric field were more concentrated in the GM and the WM for the double-cone coil compared to the Halo coil (Figures 5(c) and 5(d)) which can result in low penetration depths of the electric fields and thus less exposure of deep brain tissues to sufficient E-amplitudes. When using the HFA coil, the induced electric fields were increased over the GM and the WM surfaces mainly over the right hemisphere (Figures 5(e) and 5(f)). Numbers are provided in the Table 1 in the next section. Results for this type of coils are in agreement with those published by Lu and Ueno using the impedance method [12]. With the application of HDA coil (Figures 5(g) and 5(h)), the electric field distributions were increased over the right hemisphere and decreased in the left side compared to the HFA coil, suggesting that the penetration depth can be further improved in the right hemisphere of the brain tissues, also shown in Table 1 (next section). Electric field was further increased in the right periphery of the GW and the WM for HFA and HDA coils compared to the Halo coil configuration, which can result in further penetration depth in deep structures of the right hemisphere. The electric field was decreased in the left periphery of the GW and the WM compared to the Halo coil configuration, which can result in lower penetration depth in deep structures of the left hemisphere. As noticed for the B-field distribution, the electric field is not symmetric for HFA and HDA coils because of the asymmetrical distribution of the magnetic flux.

**Figure 6:**
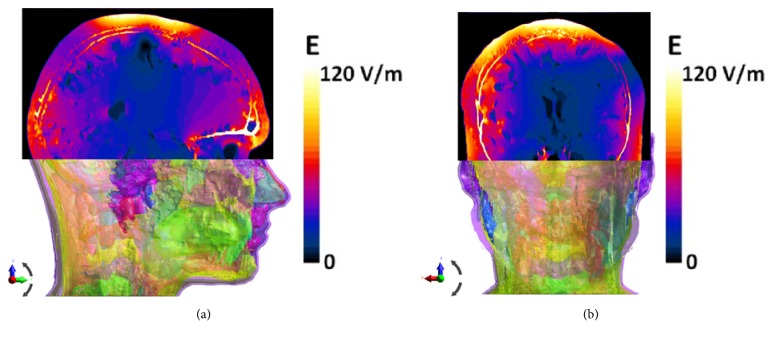
Electric field distribution (absolute value in V/m) in the cross section of MIDA model using the HDA coil. Sagittal view at x=20 cm. (b) Coronal view at y=20 cm.

Figure 6 shows the electric field distribution on cross section using the combined HDA coil. Coronal section (Figure 6(b)) shows higher electric field in the right hemisphere compared to the one in the left hemisphere for the asymmetric coil HDA, which was expected from the electric and the flux density distribution in the brain. Higher electric fields are also present in some deep structures inside the brain (at the center of Figure 6(b)). The next section will provide more quantitative evaluation of the electric field spread into deep brain structures.

### 3.3. Electric Field Spread into Deep Brain Structures

To quantify the electric field spread and penetration, Table 1 shows the percentage of volume of each tissue where the electric field amplitude is greater than half the peak of E in the cortex for each coil (V50). Due to the fact that different field distributions occur in the right and the left hemisphere of the brain tissues, percentage of volume of each brain tissue was calculated for both sides of the brain for HFA and HDA coils (double-cone and Halo coils are symmetric coils). HFA_R and HDA_R refer to the percentage of volume of each brain tissue in the right side using the HFA and HDA coils, respectively. HFA_L and HDA_L refer to the percentage of volume of each brain tissue in the left side using the HFA and HDA coils, respectively. Results show that V50 in the right hemisphere is greater than the one in the left hemisphere for the asymmetric coils, which was expected from the electric and the flux density distribution in the brain (Figures 4 and 5). This effect is more noticeable for the deeper structures like hippocampus and nucleus accumbens where the V50 is 0.04% and 1.21% in the right side of the hippocampus and nucleus accumbens, respectively, while this percentage is equal to zero in the left side (for the HDA coil). A comparison between the HDA and the HFA coils shows that a larger percentage of the right side of deep structures (hippocampus, nucleus accumbens, and cerebellum) can be reached with the HDA compared to the HFA (V50 equal to 6.2% and 3.24% for the right side of cerebellum when using the HDA and the HFA coils, respectively. Hippocampus and nucleus accumbens can only be reached when using the HDA coil with V50 equal to 0.04% and 1.21% for hippocampus and nucleus accumbens, respectively). This advantage of the HDA coil (V50 of HDA_L: 21.77%, 20.18%, and 1.94% for GM, WM, and cerebellum, respectively) over the HFA coil (V50 of HFA_L: 21.54%, 20.44%, and 1.85% for GM, WM, and cerebellum, respectively) is less important in the left side of the brain tissues. The Halo coil is targeting deeper structures in the brain (V50 equal to 2.12% for the cerebellum with the Halo coil) even without using a combined coil and spread high amplitudes of the electric field (V50 of Halo: 23.96%, 22.13%, and 2.12% for GM, WM, and cerebellum, respectively) larger than the HDA and the HFA coils in the left side of brain tissues. Double-cone (V50 of DC: 26.69% and 24.27%, for GM and WM, respectively) and Halo coils provide larger fields' distribution in the WM and the GM left side than the HDA and HFA coils due to the asymmetrical distribution of the magnetic flux. Thalamus, hypothalamus, and amygdala have 0% of V50 for each coil configuration. Gray and white matter can be reached by all coils with V50>0. Again, highest values were obtained for HFA and HDA coils.

For the purpose of deep TMS, a good coil should be characterized by a high penetration depth and high focality (i.e., a low V50). From Table 1, we can see that the double-cone coil provides better focality in the gray and white matter compared to the HDA coils in the right hemisphere (V50 equal to 26.7 and 33.8 for DC and HDA, respectively) but as a detriment of less penetration depth. In fact, the DC coil is unable to reach deeper structure like hippocampus and nucleus accumbens where the V50 of the HDA coil is equal to 0.04% and 1.21% in the right side of the hippocampus and nucleus accumbens, respectively. This depth-focality tradeoff is inherent to most of the TMS coils. Coils that are characterized by a higher penetration depth (HDA and HFA) could at the same time induce a high field amplitude in a very wide area of the cortex (Table 1). On the other hand, the coils with a more focal electric field amplitude distribution (DC and Halo) are not able to reach deep brain structures (Table 1). None of the coils proposed is able to overcome this tradeoff, as suggested also by the previous work [15] since reaching deeper brain structures implies a wider electric field spread on the cortical surface.

## 4. Conclusion

A double-cone coil combined with a Halo coil has been numerically investigated and characterized for deep brain stimulation using anatomically realistic heterogeneous head models. The 3D distribution of the B-field and the electric field were obtained for Halo, double-cone, HFA, and HDA coils. The spread of the electric fields was computed and compared for different brain tissues including deep brain tissues (thalamus, hypothalamus, amygdala, hippocampus, nucleus accumbens, and cerebellum) using Halo, double-cone, HDA, and HFA coils and showed that the asymmetrical magnetic field distribution produced by the HDA coil improved the spread of the electric field inside deep brain structures (hippocampus, nucleus accumbens, and cerebellum) and thus enabling stimulation of the brain at greater depths. Limitations of the current version of the numerical model should include the absence of the appropriate incorporation of the tissue anisotropy especially in the white matter, which would increase the model precision and could affect the electric field distribution [14]. Sensitivity of the coils' position should also be performed in future to characterize its effect of the induced fields.

## Figures and Tables

**Table 1:** Percentage of volume of each brain tissue where the amplitude of E is greater than 50% of the peak of E (V50) in the cortex for each coil configuration.

**Coil**	**Gray Matter**	**White Matter**	**Hippocampus**	**Nucleus Accumbens**	**Cerebellum**
**HFA_R**	34.04	32.34	0	0	3.24
**HDA_R**	33.84	33.07	0.04	1.21	6.20
**HFA_L**	21.54	20.44	0	0	1.85
**HDA_L**	21.77	20.18	0	0	1.94
**DC**	26.69	24.27	0	0	0
**Halo**	23.96	22.13	0	0	2.12

(i) HFA_R and HDA_R refer to the percentage of volume of each brain tissue in the right side using the HFA and HDA coils, respectively. (ii) HFA_L and HDA_L refer to the percentage of volume of each brain tissue in the left side using the HFA and HDA coils, respectively. (iii) DC refers to the double-cone coil. (iv) Thalamus, hypothalamus, and amygdala have 0% of tissue volume where the amplitude of E has 50% of the peak of E in the cortex for each coil configuration.

## Data Availability

The data used to support the findings of this study are available from the corresponding author upon request.
